# Supplementary material for: Vitamin D3 Upregulated Protein 1 Deficiency Promotes Azoxymethane/Dextran Sulfate Sodium-Induced Colorectal Carcinogenesis in Mice
Source: Cancers (Basel). 2024 Aug 23;16(17):2934. doi: 10.3390/cancers16172934 (PMC11394134; doi:10.3390/cancers16172934)
Supplement: Supplementary file 1 [file cancers-16-02934-s001.zip › cancers-3125957-supplementary.pdf]

Supplementary Information

Table S1. Disease activity index scoring criteria.

score	Body weight	Diarrhea Score	Rectal bleeding
0	No weight loss	Normal pellets	Normal
1	Weight loss of 1-5%	Slightly loose feces	Slightly bloody
2	6-10%	Loose feces	Bloody
3	11-20%	watery diarrhea	growth bleeding
4	More than 20%		

Table S2. qRT-PCR primers.

Primer	Direction	Sequence (5′→3′)
β-actin	forword	TGGAATCCTGTGGCATCCATGAAAC
	reverse	TAAAACGCAGCTCAGTAACAGTCCG
VDUP1	forword	TGGCTCCAAGAAAGTCATCC
	reverse	TTGAGAGTCGTCCACATCGT
IL-6	forword	AAAGAGTTGTGCAATGGCAATTC
	reverse	ATCTTTTACCTCTTGGTTGAAGATATGA
TNFα	forword	GCGGACTACTATGCTAAAGAGG
	reverse	GTAGAGTTCCACATGTTGCTCC
Bcl-XL	forword	CTGGGACACTTTTGTGGATCTCT
	reverse	GAAGCGCTCCTGGCCTTT
Cyclin D1	forword	TCCGCAAGCATGCACAGA
	reverse	GGTGGGTTGGAAATGAACTTCA

Table S3. Antibody list.

Company	Antibodies	Host	Experiment
Proteintech	VDUP1	Mouse	WB, IHC
Cell Signaling Technology	KI-67	Rabbit	IHC
biorbyt	p53	Rabbit	IHC
Cell Signaling Technology	β-catenin	Rabbit	IHC
Abcam	p-p65 (S536)	Rabbit	IHC
Cell Signaling Technology	p-p65 (S536)	Rabbit	WB
Cell Signaling Technology	p65	Rabbit	WB
Abcam	pSTAT3 (Y705)	Rabbit	WB
Cell Signaling Technology	STAT3	Rabbit	WB
Cell Signaling Technology	caspase-3	Rabbit	WB
Cell Signaling Technology	cyclin D1	Rabbit	WB
Cell Signaling Technology	β-actin	Rabbit	WB

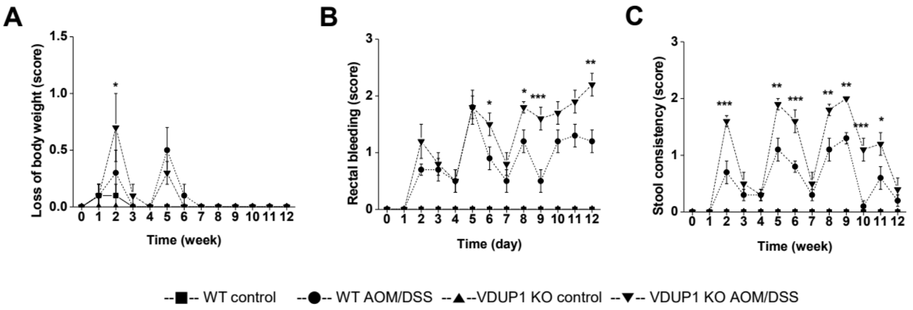

Figure S1. Vitamin D3 upregulated protein 1 (VDUP1) deficiency increases the disease severity in a mouse model of colitis-associated cancer. Wild-type (WT) and VDUP1 knockout (KO) mice were intraperitoneally injected (IP) with 7.4 mg/kg azoxymethane (AOM) on day 0, followed by three cycles of 2% dextran sulfate sodium (DSS; w/v). The mice were culled on day 85. They were scored weekly for (A) loss of body weight (B) rectal bleeding, and (C) stool consistency based on the indicated criteria (Supplementary Table S1; n=12). Data are represented as the mean ± standard error of the mean (SEM). *p<0.05, **p<0.01, and ***p<0.001.

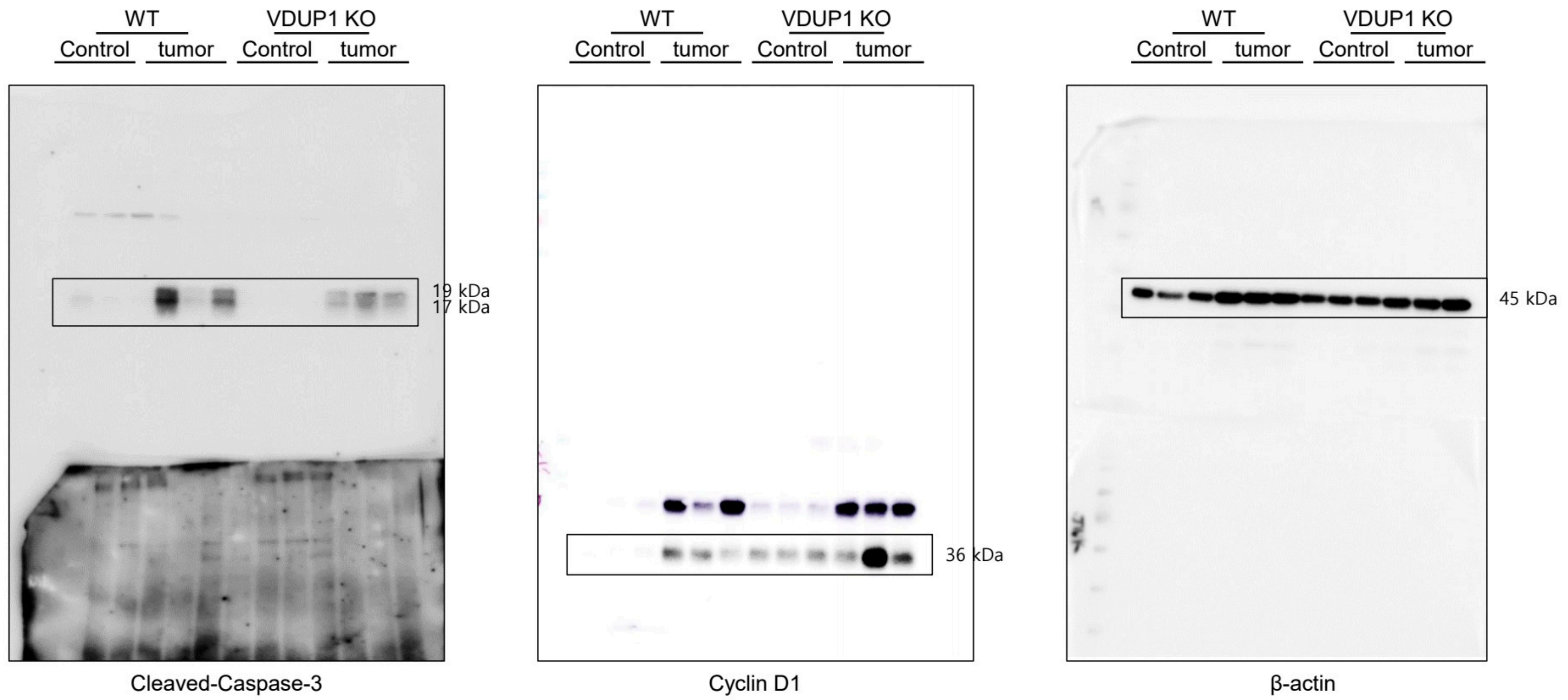

Figure S2. Original image of Fig. 5E. The black inner box indicates the cutting line of western blot images.

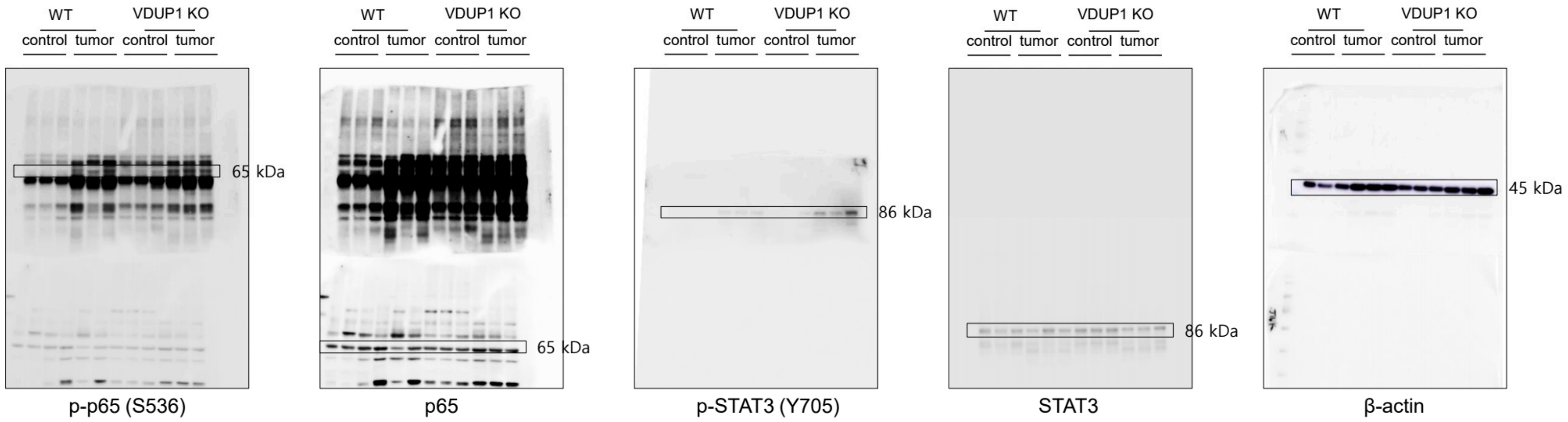

Figure S3. Original image of Fig. 6E. The black inner box indicates the cutting line of western blot images.

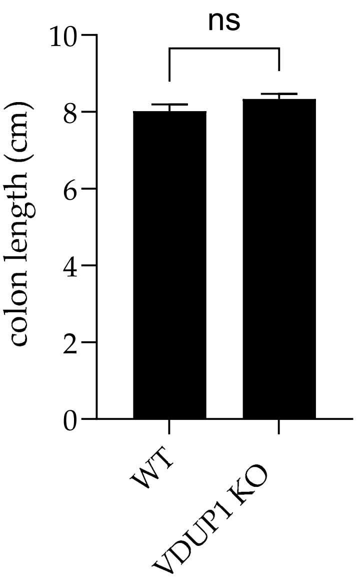

Figure S4. Colon length of the control group of WT and *VDUP1* KO mice. WT (n=13) and *VDUP1* KO (n=13) mice were intraperitoneally injected with 7.4 mg/kg AOM on day 0, followed by three cycles of 2% DSS (W/V). On day 85, the colons were resected. The Conlon length of control group of WT and *VDUP1* KO mice was determined (n=8). Data are represented as the mean ± SEM. ns, not significant.

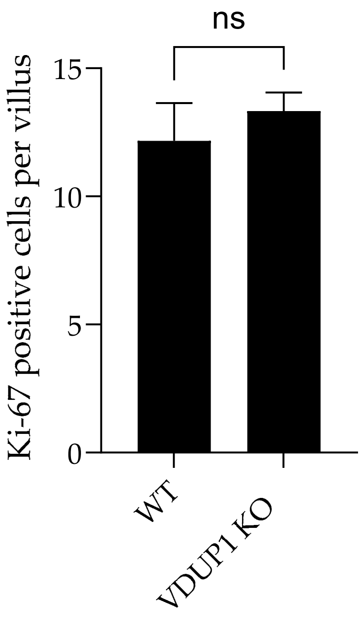

Figure S5. Ki-67 positive cells of the control group of WT and *VDUP1* KO mice. WT (n=13) and *VDUP1* KO (n=13) mice were intraperitoneally injected with 7.4 mg/kg AOM on day 0, followed by three cycles of 2% DSS (W/V). On day 85, the colons were resected. IHC staining of Ki-67 was conducted and Ki-67 positive cells were calculated in five villi per animal (n=3). Data are represented as the mean ± SEM. ns, not significant.

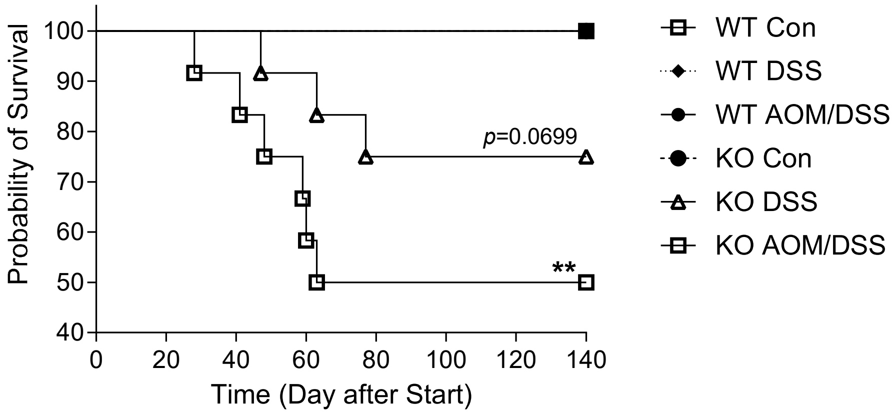

Figure S6. Ki-67 positive cells of the control group of WT and *VDUP1* KO mice. WT (n=12) and *VDUP1* KO (n=12) mice were intraperitoneally injected with 7.4 mg/kg AOM on day 0, followed by three cycles of 2% DSS (W/V). DSS groups were administered only three cycles of 2% DSS(w/v). The mortality of the experimental animals was monitored daily until day 140 after the start of the experiment. Survival curve comparing WT versus *VDUP1* KO mice. $^{**}p<0.01$.
